# Reading on the Cars

**Published:** 1872-01

**Authors:** 


### READING ON THE CARS.

The traffic in railway literature is immense. Every passenger train has its news agent, who staggers through the train distributing his magazines, books and periodicals, among the idle passengers, who are only too willing to relieve the tediousness of the jaunt by becoming absorbed in some interesting romance, furnished them by the accommodating news vender.

The passenger secures a seat by the window and then braces himself for the perusal of his book, while the light is constantly changing from the deep shade of hill or wood, to the brilliant sunshine of the open plain. This incessantly changing light keeps the muscles of the iris in constant activity, regulating the amount of light that should be admitted to the eye; for it must be known that when the light is brilliant, the pupil contracts, shutting off the superfluous rays, and when it is shaded, or when darkness has set in, the pupil becomes widely dilated, admitting more light to the chamber of the eye. When these changes are made gradually, no harm results, but, when coming rapidly from a dark passage to the bright sunlight, as is constantly occurring in a railway train, the sensation is painful, and is rendered doubly so when the eyes are directed to the white pages of a book.

Then, the constant motion and oscillations of the car, renders it impossible to hold the book in one position—its distance from the eye is constantly varying, and no matter how slight this variation may be, it is instantly compensated for by the eye, thus keeping the organ constantly employed accommodating itself to distance. This becomes fatiguing, the eyes have a sort of weary, heavy feeling, and, if the reading is persisted in, soon become "bloodshot" and painful.

We have often observed young misses, intently engaged in the perusal of some romance, while upon a rapidly moving railway train, who have only been able to finish their story with preceptable discomfort. We have noticed them rubbing their eyes, shifting their positions, and holding their books at various distances from the eye, making the greatest effort to see with eyes that have already been fatigued beyond endurance. Such practices lead to serious injury to the eyes, and it is not unfrequently the case that the oculist is called upon to prescribe for a patient who has paralysis of accommodation of the eyes, produced by reading in a railway car.

Never read for more than a few minutes, when the car is in motion, if you would avoid such a calamity.

